# Budd-Chiari syndrome due to hepatic venous web outflow obstruction: percutaneous treatment with balloon angioplasty

**DOI:** 10.1590/1677-5449.200133

**Published:** 2021-08-02

**Authors:** Patrick Bastos Metzger, Kamilla Rosales Costa, Simone Lessa e Silva, Valter Ribeiro dos Santos, Vinicius Nunes, Murilo Quadro Berbert Freire, Milton Oliveira de Albuquerque Mello

**Affiliations:** 1 Escola Bahiana de Medicina e Saúde Pública – EBMSP, Salvador, BA, Brasil.; 2 Hospital Santo Antônio, Obra Sociais Irmã Dulce – OSID, Salvador, BA, Brasil.; 3 Hospital da Bahia – HBA, Salvador, BA, Brasil.

**Keywords:** Budd-Chiari syndrome, venous thrombosis, angioplasty, endovascular procedures

## Abstract

The Budd-Chiari syndrome is a rare hepatic venous disease. It is more prevalent in young adults and may present in acute, subacute, or chronic forms, causing portal hypertension. Traditional treatment consists of thrombolysis techniques and transjugular intrahepatic portosystemic shunt, as a bridge to liver transplantation. Recently, use of balloon or stent angioplasty techniques has been reported for treatment of this condition. In this article, we report and discuss a case of BCS by membranous obstruction in the hepatic vein outflow tract, with middle hepatic vein thrombosis, in a 24-year-old patient. The treatment chosen and employed was transjugular balloon angioplasty, which achieved satisfactory results and good clinical evolution.

## INTRODUCTION

Primary Budd-Chiari syndrome (BCS) is an extremely rare hepatic vascular disease. It is caused by obstruction of venous outflow, generally at the level of the hepatic vein (HV) or the inferior vena cava (IVC).^1^^-^3 Global epidemiological data on BCS are scarce and it is estimated that incidence is around one case per million inhabitants per year, considering epidemiological studies conducted in Asia and Europe.^1^

The etiopathogenesis of BCS differs in oriental and occidental countries. In the majority of occidental countries, the principal etiology of BCS is thrombotic and, because of this, the principal forms of treatment are thrombolysis and transhepatic portosystemic shunt (TIPS), as bridges to liver transplantation. In China, and in many other oriental countries, the principal cause is venous occlusive disease caused by membranes in any hepatic venous segment up to the cavoatrial junction, and angioplasty with or without stent is the first-choice option for treatment.^2^^,^^3^

We describe endovascular treatment of a case of BCS caused by membranous occlusive of the suprahepatic outflow, with clinical and hemodynamic repercussions, conducted via transjugular venography using balloon angioplasty.

The Research Ethics Committee approved this study (decision number 4.749.464).

## CASE DESCRIPTION

**Figure 1:**
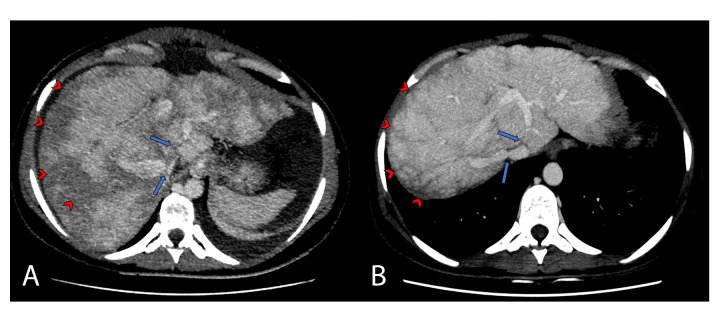
Portal phase axial tomographic images during preoperative preparation for the first endovascular intervention (A) and before the second endovascular intervention (B) (2 years and 8 months later), demonstrating development of the stenoses in the outflows of the hepatic veins (long arrows) and the change in the proportion of perfusional disorder in the hepatic parenchyma (arrowheads). Non-opacification of the middle hepatic vein is also observed.

The patient was a previously healthy, white, 24-year-old female, born and resident in Feira de Santana, BA, Brazil, with a history of abdominal distension, prostration, and an episode of hematemesis 3 months before presentation. She had no history of traumas, prior thromboses, pregnancy, abdominal pains, jaundice, hematoma, or acholic stools. She denied having any degree of Asian heredity. Clinical examination confirmed abdominal distension, with collateral abdominal veins, hepatomegaly, and ascites. Laboratory test results for hepatic enzymes, coagulation, and bilirubin were within normal limits and serum albumin was low. Ultrasonographic examination showed ascites and normal hepatopetal portal blood flow. Computed tomography (CT) with contrast showed hepatomegaly with heterogenous highlighting (known as “nutmeg” liver), hypertrophic caudate lobe, and right and left hepatic veins with contrast retention. Visibility of the middle HV was poor, suggesting thrombosis (Figure 1A). The patient was diagnosed with BCS, probably due to obstruction of the suprahepatic outflow and was referred to the interventional radiology service for venography and treatment, after upper digestive endoscopy with endoscopic sclerotherapy of esophageal varices.

**Figure 2:**
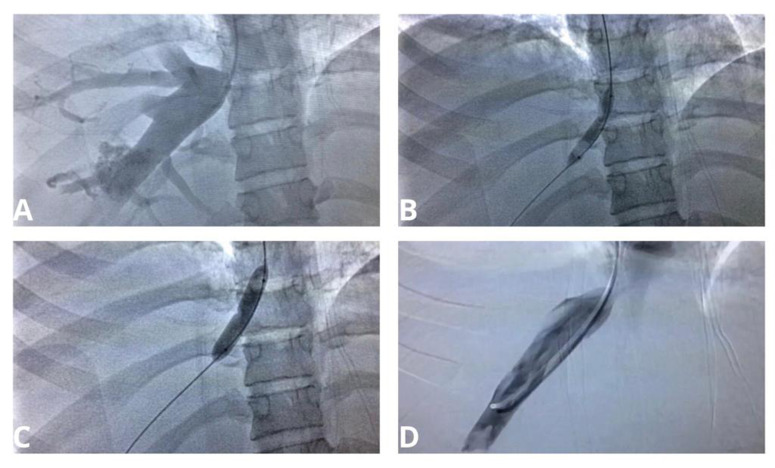
Images from intraoperative phlebography of the suprahepatic vein followed by balloon angioplasty in the first intervention. In (A), phlebography demonstrating hepatic vein stasis, caused by stenosis of the suprahepatic vein outflow. In (B), pre-dilation with an 8 mm balloon. Observe the stenosis induced in the balloon. In (C), dilation with a 12 mm balloon. Observe the reduced stenosis induced in the balloon. In (D), control phlebography demonstrating considerable improvement in outflow.

Venography was conducted under local anesthesia and analgesia with conscious sedation, using a transjugular approach, but it was difficult to catheterize the suprahepatic vein. Intravascular ultrasound (IUS) was then employed to achieve better visualization of the HV outflow into the IVC. This confirmed > 80% stenosis of the outflow of the right and left HVs and phlebography demonstrated venous stasis in both these veins, in addition to critical stenosis of the outflow. The middle HV could not be seen. A 0.035 x 260 mm hydrophilic guidewire (Terumo Medical, Somerset, NJ, United States) was then used to cross the critical suprahepatic stenosis, via the right jugular vein. Flow was reestablished by angioplasty of the stenosis using high pressure balloons, initially an 8 mm balloon and then a 12 mm balloon, (Figure 2). The patient recovered satisfactorily during the postoperative period, with considerable reductions in waist circumference and collateral abdominal vessels, and was discharged from hospital 15 days after the procedure, on rivaroxaban, which it was intended she would take for 6 months.

Postoperative follow-ups at 1, 3, and 12 months were favorable, without signs of portal hypertension syndrome (PHS) in clinical examinations, laboratory tests, or imaging exams. Tests for thrombophilia were run after withdrawal of anticoagulation, with normal results. After 2 years’ follow-up, the patient presented once more, at an ambulatory consultation, 14 weeks pregnant, with no clinical or laboratory signs of decompensation. She was put on anticoagulation with fractionated heparin by the gastroenterology/hematology service. The gestation went to full term, with delivery at 38 weeks and the patient continued to progress well in the postnatal period, still on anticoagulation. After puerperium, CT demonstrated a reduction in the outlet diameter (7 mm) compared to the previous scan (12 mm) and ascites was detected by CT, despite no clinical evidence (Figure 1B). In conjunction with the gastroenterology/hematology service, the decision was taken to perform angioplasty of the suprahepatic vein once more.

**Figure 3:**
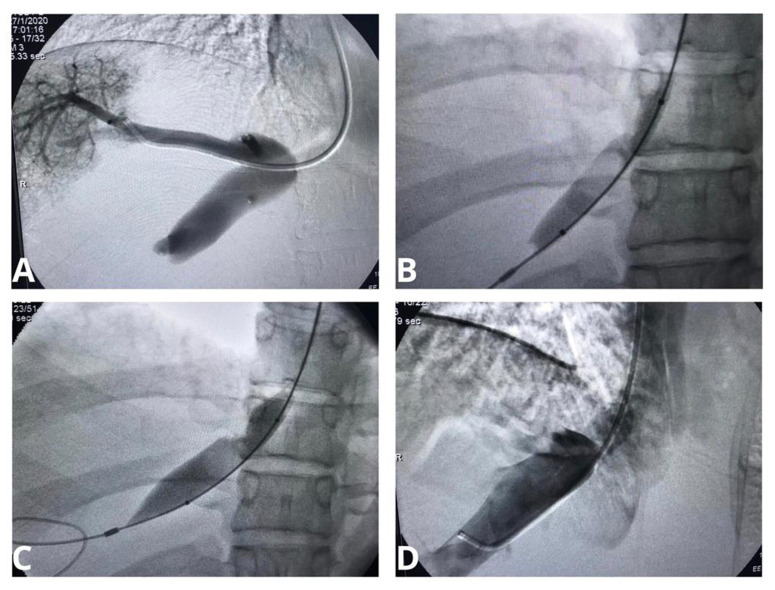
Intraoperative phlebography images of the suprahepatic vein followed by balloon angioplasty in the second intervention. In (A), phlebography demonstrating hepatic vein stasis, caused by stenosis of the suprahepatic vein outflow. In (B), pre-dilation with a 10 mm balloon. Observe the stenosis induced in the balloon. In (C), dilation with a 14mm balloon. In (D), control phlebography demonstrating considerable improvement in outflow.

The procedure was performed via the same approach, using the same anesthetic procedures described above, and phlebography showed around 50% stenosis of the outlet. Consecutive angioplasties were performed with 10 and 14 mm high pressure venous balloons. The result was satisfactory on venography and the patient was discharged from hospital the day after the procedure (Figure 3). Control CT 1 month after the procedure demonstrated an improved outlet diameter (12 mm) and absence of ascites. She is currently in follow-up, at 1 postoperative year. Total abdominal ultrasound (US) and duplex ultrasound of the suprahepatic veins are conducted every 6 months and she is asymptomatic and has normal laboratory test results. The patient gave her consent to description and publication of the case.

## DISCUSSION

Budd-Chiari syndrome is a condition with extremely low incidence. There are scant epidemiological data and the majority of studies that can be found were performed in Asia.^1^ The syndrome is caused by occlusion of hepatic venous flow at any level from the HVs to the cavoatrial junction, which can be of primary or secondary origin. Intraluminal intrinsic thrombosis is the principal primary cause of BCS. It is more frequent in young adults and those with underlying hematological abnormalities that trigger a hypercoagulable state. Conditions that cause a hypercoagulable state include myeloproliferative disorders, protein C or S deficiencies, essential thrombocytosis, systemic lupus erythematosus, factor V Leiden mutation, paroxysmal nocturnal hemoglobinuria, antiphospholipid syndrome, and use of oral contraceptives. Secondary causes can be classified as extraluminal compression (by abscess or tumor) or intraluminal invasion (tumoral or parasitic).^2^^-^5

The pathophysiology of formation of intraluminal thrombosis is not well understood, but the primary suspicion is that it is a multifactorial process involving constant respiratory movement of the diaphragm in conjunction with a perpendicular confluence of the suprahepatic vein with the IVC, causing microscopic endothelial lesions to the suprahepatic segment of the IVC. Additionally, in the occidental population diagnosed with BCS, there is a strong association with a hypercoagulable state that promotes microthrombosis. This process is slow and gradual, leading to organization of fibrotic tissue on the vein wall. In contrast, it is uncommon to detect underlying hematological abnormalities in oriental patients, highlighting the relevant differences in the etiology of the syndrome in these distinct populations, since membranous obstructions are the principal cause of BCS in oriental populations.^5^

Budd-Chiari syndrome can be classified according to time since onset and severity, as fulminant, acute, subacute, or chronic.^2^ Another classification employed is based on the site of involvement by the obstruction: the IVC type (only involves the IVC, without compromising the HV), the HV type (involves the HV, including the accessory HV, but without compromising the IVC), or the combined type (compromising the IVC and at least one of the tributaries of the HV).^5^ These classifications are important when planning the treatment approach. The case described above involved a primary obstruction, with no known underlying coagulation dysfunction, and was classified as chronic and as HV type, as shown by clinical status and by the CT with contrast.

Formation of the membranous obstruction in the interior of the vein is a slow process that is compensated for by establishment of collateral circulation. Symptoms only become apparent when stenosis is significant and patients remain asymptomatic up until this point.^5^ However, clinical presentation is also dependent on the extent and speed of obstruction of outflow and may manifest in a few days or after years of progression.^4^ Symptoms progress to liver cirrhosis, when not treated in time.^6^ When the acute form sets in, it can culminate in liver failure, manifesting with nausea, vomiting, jaundice, and laboratory findings.^2^^,^^6^ In turn, the chronic form can involve presence of hepatomegaly, jaundice, and PHS, in addition to later complications such as gastrointestinal tract bleeding and hepatocellular carcinoma.^2^^,^^4^^,^^6^

The case described involves a young, previously healthy patient with chronic complaints of abdominal distension, prostration, and episodes of hematemesis. On physical examination, she presented clinical findings of PHS. Budd-Chiari syndrome should be included on the differential diagnosis lists of all patients who exhibit these findings in the absence of primary or secondary parenchymatous liver disease.^7^

Treatment options are anticoagulation, endovascular decompression treatment (thrombolysis, angioplasty, or transjugular intrahepatic portosystemic shunt), or liver transplantation. Expansion of the treatment possibilities for BCS has enabled a considerable increase in the survival rate of these patients (80-90%).^6^

Balloon angioplasty or stenting is the first line treatment for primary BCS due to obstructive membranes, with efficacy of 95.7%.^3^^,^^6^^,^^8^^-^10 It can be performed via jugular, percutaneous hepatic, or femoral accesses, although in some cases a combined approach is needed.^3^^,^^7^^-^10 The approach route chosen for the patient described above was jugular, which has a high success rate, because the angle between the HV and the IVC is relatively large, facilitating passage of the guidewire.^6^ After the guidewire has crossed the occluded segment, the segment with stenosis is dilated using high pressure balloons with progressively larger diameters.^3^ Progressive ballooning and slow inflation and deflation of balloons should be performed in accordance with preoperative tomographic measurements and also in observance of symptoms of pain reported by the patient — no additional balloon pressure should be applied once the patient has complained of pain. Stenting is reserved for lesions in which the pressure gradient does not improve after balloon angioplasty or when there is significant recoil, even after progressive balloonings. In the case presented above, it was possible to detect improvement in clinical status soon after dilation of the stenosis, with reductions in ascites, edema, and collateral abdominal vessels.^3^

After endovascular treatment, it is necessary to monitor the patient with clinical assessments and imaging exams using duplex USG and CT,^2^ in addition to maintaining anticoagulation for at least 6 months after angioplasty, although this period can vary depending on the condition of the patient.^6^ This patient developed another stenosis 2.5 years after the initial endovascular treatment, soon after the postnatal period. Pregnancy is a physiological condition responsible for establishment of a hypercoagulable state from the 10th week of gestation onwards, which could be determinant in formation of a new membranous obstruction of the HV and consequent thrombosis.^3^^,^^11^ It was therefore necessary to introduce anticoagulation and carefully monitor the case, so that in the case of possible clinical decompensation, measures such as balloon angioplasty or stenting, in the event of failed ballooning, would be available promptly.
