# Supplementary material for: Budd-Chiari syndrome due to hepatic venous web outflow obstruction: percutaneous treatment with balloon angioplasty
Source: J Vasc Bras. 2021 Aug 2;20:e20200133. doi: 10.1590/1677-5449.200133 (PMC8336983; doi:10.1590/1677-5449.200133)
Supplement: Translation

# Translation: Síndrome de Budd-Chiari por obstrução da via de saída da veia hepática por membrana: tratamento percutâneo por angioplastia com balão

**Authors:** Patrick Bastos Metzger, Kamilla Rosales Costa, Simone Lessa e Silva, Valter Ribeiro dos Santos, Vinicius Nunes, Murilo Quadro Berbert Freire, Milton Oliveira de Albuquerque Mello

**Affiliations:** 1 Escola Bahiana de Medicina e Saúde Pública – EBMSP, Salvador, BA, Brasil.; 2 Hospital Santo Antônio, Obra Sociais Irmã Dulce – OSID, Salvador, BA, Brasil.; 3 Hospital da Bahia – HBA, Salvador, BA, Brasil.

**Keywords:** síndrome de Budd-Chiari, trombose venosa, angioplastia, procedimentos endovasculares

## Abstract

A síndrome de Budd-Chiari é uma doença venosa hepática rara, mais incidente em adultos jovens, podendo se apresentar na forma aguda, subaguda ou crônica, o que resulta em hipertensão portal. O tratamento tradicional consiste em técnicas de trombólise e de *shunts* portossistêmicos intra-hepáticos, como pontes para o transplante hepático. Recentemente, técnicas de angioplastia com balão ou stents têm sido relatadas para o tratamento dessa afecção. Neste artigo, é relatado e discutido um caso de síndrome de Budd-Chiari por obstrução membranosa da via de saída da veia supra-hepática com trombose da veia hepática média em uma paciente de 24 anos. O tratamento estabelecido foi a angioplastia transjugular com balão, que obteve resultados satisfatórios e boa evolução clínica.

## INTRODUÇÃO

A síndrome de Budd-Chiari (SBC) primária é uma doença vascular hepática extremamente rara, decorrente da obstrução do fluxo venoso de saída hepático, geralmente ao nível da veia hepática (VH) ou da veia cava inferior (VCI)^1^^-^3. Os dados epidemiológicos globais da SBC são escassos e estimam que a sua incidência seja cerca de um caso por milhão de habitantes por ano, considerando estudos epidemiológicos nos continentes asiático e europeu^1^.

A etiopatogênese da SBC difere entre países orientais e ocidentais. Na maioria dos países ocidentais, a principal etiologia da SBC é trombótica, e, por isso, as principais formas de tratamento são a trombólise e o *shunt* trans-hepático portossistêmico (TIPS), como pontes para o transplante hepático. Já na China e em diversos outros países orientais, a principal causa é a doença venosa oclusiva por membrana desde qualquer segmento venoso hepático até a junção cavo-atrial, sendo a angioplastia com ou sem stent a primeira escolha para tratamento^2^^,^^3^.

Relatamos o tratamento endovascular de uma SBC por membrana oclusiva na via de saída da veia supra-hepática, com repercussão clínica e hemodinâmica, pela venografia via acesso jugular e uso de angioplastia com balão.

O protocolo foi aprovado pelo Comitê de Ética de nossa instituição (parecer número 4.749.464).

## DESCRIÇÃO DO CASO

Paciente do sexo feminino, 24 anos, previamente saudável, raça branca, natural e procedente de Feira de Santana (BA), com história de distensão abdominal, prostração e um episódio de hematêmese havia 3 meses. A paciente não apresentava histórico de traumas, tromboses prévias, gestação, dor abdominal, icterícia, hematomas ou acolia fecal, assim como negava qualquer descendência oriental. O exame clínico confirmou a distensão abdominal, com veias colaterais abdominais, hepatomegalia e ascite. O exame laboratorial revelou enzimas hepáticas, provas de coagulação e bilirrubina dentro da normalidade e albumina sérica baixa. O exame ultrassonográfico revelou ascite com fluxo sanguíneo portal hepatopedal normal. A tomografia computadorizada (TC) com contraste revelou hepatomegalia com realce heterogêneo, denominado fígado em “noz-moscada”, lobo caudado hipertrofiado, e as veias hepáticas direita e esquerda apresentavam retenção de contraste. Não houve boa visualização da VH média, sugerindo trombose (Figura 1A). A paciente foi diagnosticada com SBC por provável obstrução da via de saída da veia supra-hepática, sendo encaminhado à radiologia intervencionista para venografia e tratamento, após endoscopia digestiva alta com escleroterapia endoscópica das varizes esofágicas.

**Figura 1:** Imagens tomográficas axiais na fase portal do pré-operatório da primeira intervenção em (A) e antes da segunda intervenção (B) endovascular (2 anos e 8 meses após), demonstrando a evolução das estenoses nas vias de saídas nas veias hepáticas (setas longas) e da alteração na proporção dos distúrbio perfusional do parênquima hepático (cabeça de seta). Nota-se também a não opacificação da veia hepática média.

A venografia foi realizada sob anestesia local e analgesia com sedação consciente, utilizando abordagem transjugular, mas houve dificuldade de cateterização da veia supra-hepática. Foi, então, utilizado o ultrassom intravascular (UI) para melhor visualização da desembocadura da VH na VCI. O UI confirmou uma estenose > 80% na via de saída das VHs, direita e esquerda, e a flebografia demonstrou estase venosa dessas duas veias, assim como estenose crítica na via de saída. A VH média não foi visualizada. Um fio guia hidrofílico 0,035 x 260 mm (Terumo Medical, Somerset, NJ) foi, então, introduzido através da estenose crítica da veia supra-hepática, via veia jugular direita. A angioplastia da estenose com balões de alta pressão de 8 mm, inicialmente, e 12 mm posteriormente restabeleceu o fluxo (Figura 2). A paciente evoluiu satisfatoriamente no pós-operatório, com acentuada redução na circunferência abdominal e dos vasos colaterais abdominais, tendo alta hospitalar com 15 dias de pós-operatório, em uso de rivaroxabana, com a intenção de uso por 6 meses.

**Figura 2:** Imagens da flebografia intraoperatória de veia supra-hepática seguida de angioplastia com balão na primeira intervenção. Em (A), flebografia demonstrando estase de veias hepáticas, ocasionada por estenose em via de saída da veia supra- hepática. Em (B), pré-dilatação com uso de balão de 8 mm. Observe a estenose ocasionada no balão. Em (C), dilatação com balão de 12 mm. Observe a diminuição da estenose ocasionada no balão. Em (D), flebografia de controle demonstrando melhora importante do fluxo na via de saída.

Durante o acompanhamento pós-operatório com 1, 3 e 12 meses, apresentou evolução favorável e sem sinais de síndrome de hipertensão portal (SHP) nos exames clínicos, laboratoriais e de imagem. Foram feitos exames para trombofilia, após a suspensão da anticoagulação, com resultados normais. Após 2 anos de acompanhamento, a paciente retornou em consulta ambulatorial grávida de 14 semanas, sem sinais de descompensação clínica ou laboratorial, sendo introduzida anticoagulação com heparina fracionada pela gastroenterologia e hematologia. A gestação veio a termo com 38 semanas, e, no puerpério, a paciente manteve evolução favorável, sendo mantida a anticoagulação. Após o puerpério, realizou nova TC que demonstrou uma diminuição do diâmetro da via de saída (7 mm) comparada ao exame prévio (12 mm), com o aparecimento de ascite detectada pela TC, porém sem evidências clínicas (Figura 1B). Optado, em conjunto com a gastro-hepatologia, por nova angioplastia da veia supra-hepática.

O procedimento foi realizado pela mesma via e utilizando os procedimentos anestésicos anteriormente descritos, sendo evidenciada estenose na via de saída de cerca de 50% pela flebografia. Foram realizadas angioplastias consecutivas com balões venosos de alta pressão de 10 e 14 mm. O resultado venográfico foi satisfatório, e a alta hospitalar foi realizada no dia seguinte ao procedimento (Figura 3). A TC de controle com 1 mês de pós-operatório demonstrou uma melhora do diâmetro da via saída (12 mm) e ausência de ascite. Atualmente encontra-se em acompanhamento, com 1 ano de pós-operatório, sendo realizado ultrassom abdominal total (USG) e dúplex das veias supra-hepáticas a cada 6 meses, estando assintomática e com exames laboratoriais normais. A paciente deu consentimento para a descrição e publicação do caso.

**Figura 3:** Imagens da flebografia intraoperatória de veia supra-hepática seguida de angioplastia com balão na segunda intervenção. Em (A), flebografia demonstrando estase de veias hepáticas, ocasionada por estenose em via de saída da veia supra- hepática. Em (B), pré-dilatação com uso de balão de 10 mm. Observe a estenose ocasionada no balão. Em (C), dilatação com balão de 14 mm. Em (D), flebografia de controle demonstrando melhora importante do fluxo na via de saída.

## DISCUSSÃO

A SBC constitui uma afecção de incidência extremamente baixa. Os dados epidemiológicos são escassos, e, quando encontrados, os estudos são em sua maioria realizados no continente asiático^1^. Essa síndrome é causada pela oclusão do fluxo venoso hepático a qualquer nível desde as VHs até a junção átrio-caval, podendo ser de origem primária ou secundária. A trombose intraluminal intrínseca constitui a principal causa primária da SBC, mais frequente em adultos jovens e com anormalidade hematológica subjacente, que desencadeia um estado de hipercoagulabilidade. Algumas das afecções que originam o estado de hipercoagulação são doença mieloproliferativa, deficiência de proteína C ou S, trombocitose essencial, lúpus eritematoso sistêmico, mutação do fator V Leiden, hemoglobinúria paroxística noturna, síndrome antifosfolipídica e uso de contraceptivos orais. Já as causas secundárias podem ser representadas por compressão extraluminal (abscesso ou tumor) ou ainda por invasão intraluminal (tumoral ou parasitária)^2^^-^5.

A fisiopatologia da formação da trombose intraluminal não é bem conhecida, mas a principal suspeita é de um processo multifatorial que envolve o constante movimento respiratório do diafragma associado a uma confluência perpendicular da veia supra-hepática à VCI, que causa lesões endoteliais microscópicas na VCI em seu segmento supra-hepático. Além disso, na população ocidental diagnosticada com SBC, é possível observar uma forte associação com um estado de hipercoagulabilidade que promove a microtrombose. Esse processo é lento e gradual, levando a uma organização de tecido fibrótico na parede da veia. Já nos pacientes orientais, é incomum identificar anormalidades hematológicas subjacentes, evidenciando diferenças relevantes da etiologia dessa síndrome entre as distintas populações, sendo a presença de membranas a principal causa para a SBC nessa população^5^.

A SBC pode ser classificada de acordo com o seu tempo de evolução e gravidade, em fulminante, aguda, subaguda ou crônica^2^. Outra classificação utilizada é de acordo com o local de acometimento da obstrução: tipo VCI (acomete apenas a VCI, sem comprometimento da VH), tipo VH (acometimento da VH, incluindo a VH acessória, mas sem comprometimento da VCI) e tipo combinado (comprometimento da VCI e pelo menos uma das tributárias da VH)^5^. Essas classificações são importantes no planejamento da abordagem terapêutica. O caso relatado trata-se de uma obstrução primária, sem disfunção de coagulabilidade subjacente conhecida, classificada como crônica e do tipo VH, como evidenciado pelo quadro clínico e pela TC com contraste.

A formação da membrana obstrutiva no interior da veia é um processo lento que apresenta compensação através do estabelecimento de circulação colateral. Os sintomas só se tornam presentes nos pacientes quando a estenose é significativa, permanecendo assintomático até esse momento^5^. No entanto, a apresentação clínica depende ainda da extensão e rapidez da obstrução do fluxo de saída, podendo manifestar-se em poucos dias ou após anos de evolução^4^. Os sintomas são progressivos até cirrose hepática, quando não tratada em tempo hábil^6^. Quando instaurado o quadro agudo, pode culminar em insuficiência hepática, manifestando-se com náuseas, vômitos, icterícia e alterações laboratoriais^2^^,^^6^. Já o quadro crônico pode caracterizar-se pela presença de hepatomegalia, icterícia e SHP, além de complicações mais tardias, como sangramento do trato gastrointestinal e carcinoma hepatocelular^2^^,^^4^^,^^6^.

O caso relatado evidencia uma paciente jovem, previamente saudável, com queixas crônicas de distensão abdominal, prostração e um episódio de hematêmese. Ao exame físico, apresentou os achados clínicos de SHP. A SBC deve constituir um dos diagnósticos diferenciais para todo paciente que apresentar esses achados na ausência de doença parenquimatosa hepática primária ou secundária^7^.

As opções de tratamento são anticoagulação, terapia endovascular de descompressão (trombólise, angioplastia ou *shunt* portossistêmico intra-hepático transjugular) ou transplante de fígado. A evolução das possibilidades de tratamento para a SBC possibilitou um importante aumento da sobrevida desses pacientes (80-90%)^6^.

A angioplastia com balão ou stent é a primeira linha para o tratamento da SBC primária por membrana obstrutiva, e sua eficácia é de 95,7%^3^^,^^6^^,^^8^^-^10. Pode ser realizada por via jugular, hepática percutânea ou femoral; no entanto, em alguns casos se faz necessária a abordagem combinada^3^^,^^7^^-^10. A via de acesso escolhida para a abordagem da paciente em questão foi a via jugular, que apresenta alta taxa de sucesso, pois o ângulo entre a VH e a VCI é relativamente grande, facilitando a passagem do fio-guia^6^. Após a passagem do fio-guia pelo segmento ocluído, é realizada a dilatação do segmento estenosado com o uso do balão de alta pressão com diâmetros progressivamente maiores^3^. O uso do balonamento progressivo, assim como a insuflação e desinsuflação lenta dos balões, deve ser realizado consoante às medidas tomográficas pré-operatórias e também conforme a sintomatologia de dor da paciente — uma vez havendo essa queixa, pressão maior no balão não deve ser alcançada. O uso do stent é reservado para lesões que não melhoram o gradiente pressórico com a angioplastia com balão ou que apresentam *recoil* importante, mesmo após balonamentos progressivos. No caso apresentado, após a dilatação do segmento estenosado, foi possível notar a melhora do quadro clínico de forma precoce, com redução da ascite, do edema e dos vasos colaterais abdominais^3^.

Após o tratamento endovascular, faz-se necessário o acompanhamento do paciente com avaliação clínica e realização de exames de imagem com USG com Doppler e TC^2^, além de manutenção de anticoagulação por pelo menos 6 meses após a angioplastia, podendo esse período ser variável de acordo com as condições do paciente^6^. A paciente apresentou uma nova estenose após 2,5 anos do tratamento endovascular inicial, logo após o período de puerpério. Essa condição fisiológica é responsável pelo estabelecimento de um estado de hipercoagulabilidade a partir da 10ª semana de gestação, o que pode agir como determinante na formação de nova membrana obstrutiva da VH e consequente trombose^3^^,^^11^. Portanto, tornam-se necessários a anticoagulação e o acompanhamento rigoroso do caso, para que, em uma eventual descompensação clínica, medidas como nova angioplastia com balão ou com stent, em caso de falha, estejam prontamente disponíveis.
